# Iron-sensing is governed by mitochondrial, not by cytosolic iron–sulfur cluster biogenesis in *Aspergillus fumigatus*†
†Electronic supplementary information (ESI) available. See DOI: 10.1039/c8mt00263k


**DOI:** 10.1039/c8mt00263k

**Published:** 2018-11-05

**Authors:** Matthias Misslinger, Beatrix E. Lechner, Katharina Bacher, Hubertus Haas

**Affiliations:** a Division of Molecular Biology, Biocenter , Medical University of Innsbruck , Innrain 80 , 6020 Innsbruck , Austria . Email: hubertus.haas@i-med.ac.at

## Abstract

Microorganisms have to adapt their metabolism to the requirements of their ecological niche to avoid iron shortage as well as iron toxicity. Therefore, mechanisms have been evolved to tightly regulate iron uptake, consumption, and detoxification, which depend on sensing the cellular iron status. In the facultative anaerobic yeast *Saccharomyces cerevisiae*, iron-sensing depends on mitochondrial (ISC) but not cytosolic iron–sulfur cluster assembly (CIA), while in mammals further processing of an ISC product *via* CIA is required for sensing of the cellular iron state. To address the question of how the obligatory aerobic mold *Aspergillus fumigatus* senses the cellular iron state, mutant strains allowing the downregulation of ISC and CIA were generated. These studies revealed that: (i) Nfs1 (Afu3g14240) and Nbp35 (Afu2g15960), which are involved in ISC and CIA, respectively, are essential for growth; (ii) a decrease in ISC (Nfs1 depletion) but not CIA (Nbp35 depletion) results in a transcriptional iron starvation response, (iii) a decrease in, ISC as well as CIA, increases the chelatable iron pool, accompanied by increased iron toxicity and increased susceptibility to oxidative stress and phleomycin. In agreement with ISC being essential for iron-sensing, a decrease in mitochondrial iron import by deletion of the mitochondrial iron importer MrsA resulted in an iron starvation response. Taken together, these data underline that iron-sensing in *A. fumigatus* depends on ISC but not CIA. Moreover, depletion of the glutathione pool *via* generating a mutant lacking γ-glutamylcysteine synthase, GshA (Afu3g13900), caused an iron starvation response, underlining a crucial role of glutathione in iron-sensing in *A. fumigatus*.

## Abstract 2

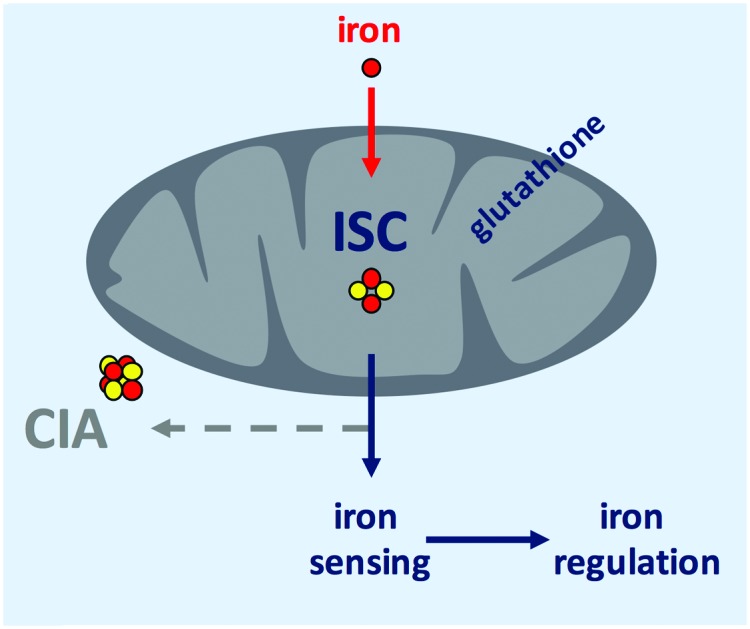
Microorganisms have to adapt their metabolism to the requirements of their ecological niche to avoid iron shortage as well as iron toxicity.

##

Significance to metallomics

Iron is an essential trace element and its sensing is crucial for virulence of the fungal pathogen *Aspergillus fumigatus*. By analyzing the impact of mitochondrial- and cytosolic iron–sulfur cluster assembly, mitochondrial iron import, and glutathione, this study gives new insights into the cellular components involved in iron-sensing.

## Introduction

*Aspergillus fumigatus* is a saprophytic fungus, spread around the world, usually growing on decaying organic matter. Nevertheless, this mold is the most common airborne fungal pathogen able to cause life-threatening invasive- and allergic diseases.^1^

Iron is an essential trace element for virtually all organisms and *A. fumigatus* has to cope with the availability of this metal in these different ecological niches to supply its metabolism with iron. On the other hand, excessive iron uptake is toxic as free iron causes the production of highly reactive hydroxyl radicals *via* the Haber–Weiss/Fenton reaction.^2^,^3^ Therefore, a sophisticated system regulating iron uptake and detoxification has been developed to keep the balance between iron deficiency and iron toxicity: during conditions of iron excess, the GATA-type transcription factor SreA represses iron uptake to avoid iron overload;^4^ during iron starvation, the bZip-type transcription factor HapX represses iron-consuming and detoxifying pathways and activates iron-acquisition genes;^5^,^6^ during conditions of iron excess, for example during a shift from iron starvation to iron sufficiency, HapX switches from a repressor of iron-consuming and iron-detoxifying genes to an activator of the very same genes.^7^ These genes include *cccA*, encoding a transporter mediating vacuolar iron deposition, which has been shown to represent the major iron detoxification strategy of *A. fumigatus*.^8^ In iron acquisition by *A. fumigatus* siderophores play a major role.^9^ Siderophores are low molecular mass ferric iron chelators, which are secreted during iron starvation. Subsequent to binding environmental iron, the ferric siderophore complex is taken up by specific transporters. The major siderophore of *A. fumigatus* is triacetylfusarinine C (TAFC). Murine aspergillosis models demonstrated that siderophore biosynthesis and HapX are essential for the pathogenicity of *A. fumigatus*,^6^,^10^ revealing that adaptation to iron starvation is an important virulence determinant of *A. fumigatus*.

Iron-mediated gene regulation depends on accurate sensing of the cellular iron status. In *Saccharomyces cerevisiae* and mammals, iron-sensing has been shown to be mediated by sensing iron–sulfur cluster (FeS) biosynthesis rather than elemental iron.^11^–^14^ FeS clusters are small inorganic cofactors consisting of iron and sulfur in various conformations, of which the chemically simplest are the rhombic [2Fe–2S]- and the cubane [4Fe–4S] types.^15^ FeS clusters exert essential functions in numerous proteins, which usually coordinate FeS *via* cysteinyl or histidinyl residues. FeS are required as cofactors for proteins involved in electron transfer, catalysis, and the regulation of gene expression. Some examples of FeS-requiring pathways are the respiratory chain (complexes I, II, and III), the citric acid cycle (*e.g.*, aconitase succinyl-CoA dehydratase), DNA replication and repair (various DNA polymerases and primases), translation (Rli1), heme biosynthesis (ferrochelatase), and the sensing of oxygen, nitric oxide, and iron.^14^,^16^–^21^

**Fig. 1:**
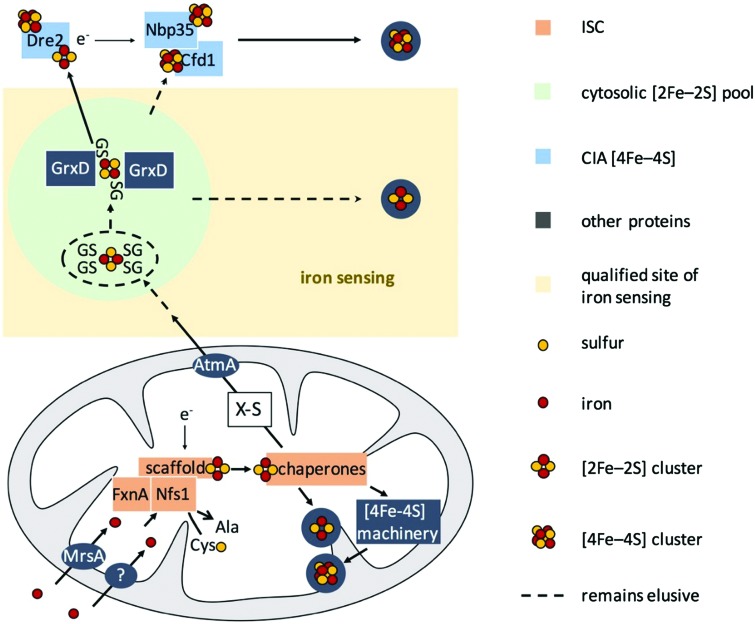
Model for FeS biosynthesis and iron-sensing in *A. fumigatus* based on current eukaryotic models focusing mainly on the components analyzed in this study. FeS biosynthesis starts with the import of iron into mitochondria *via* MrsA, the homolog of yeast Mrs3/4. Sulfur is provided by the desulfurase Nfs1 from the substrate cysteine. Employing a sophisticated cascade of scaffold proteins, electron donors, and chaperones, iron and sulfur are complexed to [2Fe–2S] clusters. This pathway is designated as ISC. In mitochondria, these [2Fe–2S] clusters are either used directly or further processed to generate [4Fe–4S] clusters. Furthermore, a sulfur-containing compound, designated as X–S, is exported from the mitochondria to fuel cytosolic iron–sulfur cluster assembly (CIA) *via* the ABC transporter AtmA, the homolog of yeast Atm1. This hitherto very loosely defined compound X–S has been proposed to be a GSH-complexed [2Fe–2S] cluster ((GS)_4_–[2Fe–2S]), possibly forming a cytosolic [2Fe–2S] cluster pool. In yeast, the cytoplasmic trafficking of [2Fe–2S] clusters has been demonstrated to involve the monothiol glutaredoxin Grx3/4, which targets [2Fe–2S] clusters to client proteins. Besides the possibility of the existence of a *de novo* assembly of FeS in CIA, the suggested pool of [2Fe–2S] clusters might be the source that fuels CIA to generate [4Fe–4S] clusters. In this study, we show that iron-sensing depends on ISC but not CIA, *i.e.*, a signal exported from the mitochondria, or in other words most likely X–S.

*S. cerevisiae* has been the role model for the characterization of eukaryotic FeS biogenesis, in which most aspects of this process seem to be highly conserved in all eukaryotes (for a review see ref. 15 and 22). A simplified scheme of eukaryotic FeS biosynthesis concerning the components dealt with in this study is shown in Fig. 1. The biosynthesis of FeS starts with the core iron–sulfur cluster assembly machinery (ISC), which is located in mitochondria. ISC depends on the import of iron into the mitochondria; the only transporter mediating mitochondrial iron import characterized in *A. fumigatus* is termed MrsA, which is homologous to *S. cerevisiae* Mrs3/4.^23^,^24^ The desulfurase Nfs1 converts cysteine to alanine by cleaving off sulfur. The sulfur is subsequently complexed with iron employing a sophisticated cascade of scaffold proteins and chaperones to form mitochondrial [2Fe–2S] clusters. Subsequently, the machinery splits up to generate mitochondrial [4Fe–4S] clusters, which are required as cofactors for *e.g.*, aconitase, lipoate synthase, and respiratory complex I & II.^25^ In a second pathway, a sketchy characterized sulfur-containing component, designated X–S, is exported from the mitochondria for cytoplasmic iron–sulfur cluster assembly (CIA).

CIA starts with the assembly of [4Fe–4S] clusters on Nbp35 and Cfd1.^17^,^26^–^30^ This reaction is assisted by the supply of electrons by Dre2. In subsequent pathways, the [4Fe–4S] cluster are transferred to cytosolic and nuclear client proteins.^15^,^31^–^33^ The link between ISC and CIA has still not been completely elucidated, but it has been shown in *S. cerevisiae* that X–S is transported to the cytoplasm *via* the ABC transporter termed Atm1 or AtmA in *A. fumigatus*.^34^,^35^ Moreover, it has been shown that glutathione (GSH) complexed [2Fe–2S] clusters ((GS)_4_–[2Fe–2S]) are a possible substrate for Atm1 *in vitro*.^36^ The monothiol glutaredoxin Grx3/4 (homolog of *A. fumigatus* GrxD) has been demonstrated to be involved in iron sensing and the cytoplasmic trafficking of [2Fe–2S] in *S. cerevisiae* and *Schizosaccharomyces pombe*.^37^,^38^

Taken together, FeS biosynthesis in *S. cerevisiae* displays a strict spatial separation of the cytosolic CIA and the independent mitochondrial ISC, which fuels CIA with the generated compound X–S.

GSH has been shown to be crucial for iron homeostasis. GSH is not only required as a redox buffer or for the biotransformation of harmful substances, it is also involved in iron-dependent regulatory circuits in *S. cerevisiae* and *S. pombe*^39^,^40^ and also in FeS assembly itself.^17^,^41^–^43^ This emerging role of GSH was reviewed recently by Berndt and Lillig.^44^ Remarkably, GSH has the capacity to coordinate iron and FeS, respectively. Therefore GSH is most likely involved in the formation of the labile iron pool (LIP).^44^ LIP is defined as the chelatable redox-active iron and is therefore regarded as the crossroad of cellular iron traffic.^45^

Several studies have indicated that, for iron homeostasis maintenance, *S. cerevisiae* senses a signal produced by ISC, most likely [2Fe–2S] clusters, rather than atomic iron or signals produced by the CIA.^26^,^46^–^50^ One of the underlying key experiments demonstrated that the downregulation of ISC, but not CIA, results in an iron starvation response mediated by the iron-regulatory transcription factors Aft1 and Aft2.^51^ Similarly, ISC was found to be crucial for the repression of iron uptake in *Candida albicans*, *S. pombe*, and *Cryptococcus neoformans*.^52^–^56^ However, these studies did not clarify the role of CIA in iron sensing in these species as an impairment of ISC also impairs CIA due to the metabolic dependence of CIA on ISC. In contrast to *S. cerevisiae*, mammalian iron metabolism is regulated by the cytosolic iron-regulatory protein IRP1, which senses the cellular iron status *via* the binding of a CIA-derived [4Fe–4S] cluster, which depends on the ISC functionality.^21^,^30^,^33^,^57^

In this study, we raised the question how *A. fumigatus* senses iron. Like *S. cerevisiae*, *A. fumigatus* belongs to the *Ascomycetes* but exhibits significant differences. In contrast to the facultative anaerobic yeast *S. cerevisiae*, which can survive without mitochondrial DNA,^58^*A. fumigatus* is an obligate aerobic multicellular organism. Moreover, *A. fumigatus* lacks homologs of the *S. cerevisiae* iron regulators Aft1/2, while the iron-regulatory transcription factors employed by *A. fumigatus*, SreA and HapX, are conserved in most fungal species except *S. cerevisiae* and the closely related *Saccharomycotina*.^12^,^59^ Furthermore, in contrast to *S. cerevisiae*, *A. fumigatus* produces siderophores for iron acquisition as well as for the storage and intracellular transport of iron.^60^

Here we show that, despite the significant differences in lifestyle and transcription factors employed for iron-regulation, iron-sensing in *A. fumigatus* depends on ISC but not on CIA, similar to *S. cerevisiae*. Moreover, we demonstrate a crucial role for GSH in the iron-sensing of *A. fumigatus*.

## Experimental

### Strains, growth conditions, and primers used

Growth assays were performed in *Aspergillus* Minimal Medium (MM) per L: 10 g glucose, 20 mM glutamine, salt solution, and iron-free trace elements according to ref. 61. For iron starvation (–Fe) conditions, no iron was added. Iron (FeSO_4_) was added separately to a final concentration of 30 μM to set up iron replete conditions (+Fe). Other iron concentrations are indicated separately in the respective figures. *pxylP*-driven genes were repressed unless xylose was added to the medium; the used xylose concentrations are indicated in the respective figures. For solid growth, the medium was solidified with 1.8% agarose. The fungal strains used in this study are summarized in Table S1 (ESI†). The fungal background was AfS77, if not otherwise declared. The oligonucleotide primers used are summarized in Table S2 (ESI†). All sequences were downloaded from ; FungiDB.org.^62^,^63^

### Depletion of Nbp35, Nfs1, or GSH

In phase one, 10^8^ spores (wildtype (wt), *nbp35*^*xylP*^, *nfs1*^*xylP*^, Δ*gshA* or *gshA*^*rec*^) were shaken in 50 mL MM +Fe at 25 °C with 0.1% xylose or 5 mM GSH, respectively, for 20 h. Germlings were centrifuged and washed once with water to remove iron and xylose or GSH before being re-suspended in 100 mL MM containing no xylose or GSH. To deplete the already produced protein or GSH in phase two, growth was continued for 20 h at 37 °C. During phase two, the growth conditions were –Fe, +Fe, sFe, or the addition of TAFC for the iron measurements (see below).

### Generation of mutant strains

For the promoter exchange cassette of *nbp35* (Afu2g15960) and *nfs1* (Afu3g14240), plasmids were assembled by using GeneArt® Seamless Cloning and Assembly Kit (Thermo) and a pUC19 backbone. 5′- and 3′-homologous regions were amplified from genomic DNA, with hygromycin amplified from plasmid pAN7-1.^64^ The xylose-inducible promoter (_*p*_*xylP*) was from the *xylP* of *Penicillium chrysogenum*.^65^ Linearized pUC19 (pUC19L) and fragments, which were amplified with the primer pairs nbp35_5′f/nbp35_5′r, hph1_f/hph1_r, xylP1_f/xylP1_r, and nbp35_3′f/nbp35_3′r were assembled to generate pMMHL15. Cassette *hph*-_*p*_*xylP* was amplified from pMMHL15 with the primers hph2_f/xylP2_r and assembled with linearized pUC19 and homologous regions for *nfs1* amplified with the primers nfs1_5′f/nfs1_5′r and nfs1_3′f/nfs1_3′r, respectively, to receive pMMHL25.

To generate a *gshA* (Afu3g13900), flanking homologous sites of *gshA* were amplified with the primers gshA_5′f/gshA_5′r and gshA_3′f/gshA_3′r and cut with PstI or HindIII, respectively. These homologous sites were ligated to a pyrithiamine resistance cassette excised from plasmid pSK275 (syn. pME3024^66^) with PstI and HindIII.

The final constructs were amplified from pMMHL15 with nbp35_5′f and nbp35_3′r, from pMMHL25 with primers nfs1_5′f and nfs1_3′r and from the Δ*gshA* deletion construct ligation with gshA_5′nest and gshA_3′nest and each transformed into an AfS77 wt recipient strain in a protoplast mediated manner^67^ employing homologous recombination.

The plasmid for the reconstitution of *gshA* was generated by amplifying the *gshA* promoter, coding sequence, and terminator-containing region with primers gshA_f/gshA_r and a hygromycin cassette with primers hph4_f/hph4_r with subsequent seamless assembly in a pUC19L plasmid. The plasmid was integrated at the *gshA* locus in a *gshA*-deficient strain to reconstitute the function of the gene by employing the 5′ flanking region for homologous recombination.

### Nucleic acid isolation, Northern blot analysis, Southern blot analysis

RNA was isolated using TRI Reagents (Sigma) according to the manufacturer's manual. 10 mg of RNA was used for electrophoresis on 2.2 M formaldehyde agarose gels and subsequently blotted onto Amersham™ Hybond™-N Membranes (ThermoFisher). Transcripts of interest were detected with DIG labeled probes amplified by PCR.

DNA was isolated by PCI extraction and isopropanol precipitation. To confirm the gene-specific restriction pattern of the genetic manipulations, DNA was digested with restriction enzymes specific for the respective gene. The resulting restriction fragments were separated on an agarose gel and transferred to Amersham™ Hybond™-N Membranes (ThermoFisher) by capillary blotting with NaOH. Signals for correct integration were detected with DIG labeled probes amplified by PCR.

### TAFC-iron uptake and intracellular siderophore measurements

To measure the uptake of TAFC-chelated iron (TAFC^+Fe^), liquid cultures were supplemented with 100 mM TAFC^+Fe^ as the sole iron source. This supplementation was done in phase two of protein or GSH depletion (see above). TAFC^+Fe^ has a reddish color and its concentration in a solution can thereby be measured by photometric means. TAFC^+Fe^ concentrations were measured in the supernatant at the beginning of growth and after harvesting the mycelia. The difference in concentration represented the consumed iron amount as 1 molecule TAFC^+Fe^ chelates one iron ion. To isolate TAFC^+Fe^ from the supernatant, it was mixed vigorously with 0.2 volumes of chloroform. The chloroform phase containing TAFC^+Fe^ was separated and mixed with 1 volume water and 5 volumes diethyl ether. The resulting upper phase containing chloroform and diethyl ether phase was removed and TAFC^+Fe^ was measured in the aqueous phase using a molar extinction factor of *ε* = 2996 M^–1^ cm^–1^ at 440 nm.^6^

To measure the intracellular siderophores, mycelia were harvested and lyophilized. 50 mg of each mycelium was ground and dissolved in 1 mL sodium-phosphate buffer (50 mM, pH 7.5). The cell debris was removed by centrifugation and the supernatant was mixed with 0.25 volume of phenol : chloroform:isoamyl alcohol (25 : 24 : 1, PCI). The PCI phase containing intracellular siderophores was separated and mixed with 1 volume water and 5 volumes diethyl ether. The resulting upper phase containing PCI and the diethyl ether phase was removed and the intracellular siderophores were measured in the aqueous phase using a molar extinction coefficient of *ε* = 2460 M^–1^ cm^–1^ at 434 nm.^68^,^69^

### Determination of the BPS-chelatable iron pool

For determination of the chelatable iron pool, we applied a method based on the chelator bathophenanthrolinedisulfonate (BPS).^70^ BPS is a ferrous iron chelator, which turns pink upon binding to Fe(ii), allowing photometric quantification of ferrous iron. To convert chelatable iron into the ferrous form and to visualize it, 1 mM ascorbic acid as a reducing agent and BPS were added to the samples to a final concentration of 1 mM. Fe(BPS)_3_. Photometric quantification was done at 535 nm using a molar extinction coefficient of *ε* = 17 000 L mol^–1^ cm^–1^.^71^

### Phleomycin and hydrogen peroxide resistance determination

To measure the effect of H_2_O_2_ or phleomycin, respectively, the minimum inhibitory concentration (MIC) was determined by serial dilution in 96-well plates.^72^ Per well, 10^4^ spores were inoculated at low induced _*p*_*xylP* conditions (xylose concentration is indicated at the respective figures). The respective inhibitor was added in serial two-fold dilution. The readout was defined as the minimal concentration that completely inhibits growth.

## Results and discussion

### nfs1 and nbp35 are essential genes

**Fig. 2:**
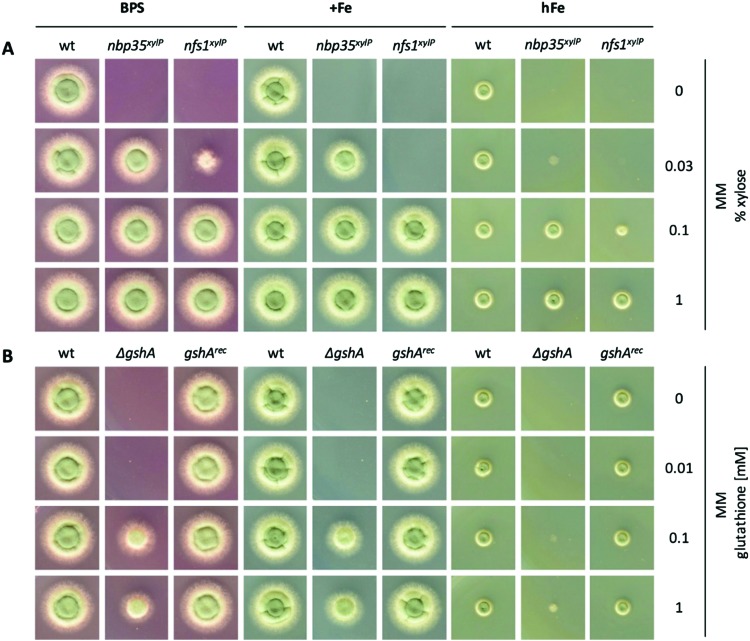
Depletion of ISC or CIA as well as showing GSH decreases iron resistance. For plate growth assays under different iron availability, 10^4^ conidia were spotted and growth was scored after incubation for 48 h at 37 °C. For iron starvation (BPS), the addition of iron was omitted and BPS was added to a final concentration of 0.2 mM. Iron sufficient medium (+Fe) contained 0.03 mM FeSO_4_, and high-iron medium (hFe) contained 10 mM FeSO_4_. (A) wt, *nbp35*^*xylP*^ and *nfs1*^*xylP*^ strains were grown on MM with different xylose concentrations for tuning _*p*_*xylP*-mediated gene expression. (B) wt, Δ*gshA*, and *gshA*^*rec*^ were grown with different GSH concentrations to rescue GSH auxotrophy of Δ*gshA* to a different degree.

It has been shown previously in other organisms that both ISC and CIA are essential pathways.^73^,^74^ The proteins involved in the ISC and CIA of *S. cerevisiae* are highly conserved in *A. fumigatus*, indicating similar functions (Table S3, ESI†). To characterize iron-sensing, we generated strains allowing the downregulation of the key components of ISC and CIA, respectively, because we expected a lethality of gene deletions. As CIA metabolically depends on ISC (Fig. 1),^75^ the downregulation of ISC leads to the downregulation of both ISC and CIA. Therefore, we generated the strains *nfs1*^*xylP*^ and *nbp35*^*xylP*^, in which *nfs1* (essential for mitochondrial ISC and consequently also CIA) or *nbp35* (essential for cytoplasmic CIA), respectively, were under the control of the xylose-inducible promoter _*p*_*xylP*, derived from the β-1,4-endoxylanase-encoding *xylP* gene of *P. chrysogenum*.^65^ Expression driven by _*p*_*xylP* promoter allows xylose concentration-dependent gene induction, while the absence of xylose represses gene expression, which leads to an effect similar to gene deletion.^8^,^76^,^77^ After confirmation of the correct integration of the constructs by Southern analysis (Fig. S1, ESI†), growth assays were performed at different promoter-induction levels. At non-inducing conditions, both mutant strains failed to grow, demonstrating the essentiality of both *nfs1*^*xylP*^ and *nbp35*^*xylP*^ for cell viability. Under full-inducing conditions (1% xylose), both mutant strains displayed a wildtype (wt)-like growth pattern under iron starvation (in the presence of the ferrous iron-specific chelator bathophenanthrolinedisulfonate (BPS)), iron sufficiency (0.03 mM, +Fe), and iron excess (10 mM, hFe), proving the functionality of the _*p*_*xylP* expression system (Fig. 2A). During iron starvation, the *nfs1*^*xylP*^ strain required a higher xylose concentration to reach full growth compared to the *nbp35*^*xylP*^ mutant (0.1% *versus* 0.03%), indicating the requirement for a higher expression level. Remarkably, both *nfs1*^*xylP*^ and *nbp35*^*xylP*^ strains displayed decreased iron resistance, *i.e.*, the medium xylose concentration (0.03% and 0.1%) did not support the same degree of growth during iron excess compared to iron sufficiency or iron starvation. This already pointed toward a possible function of FeS cluster biosynthesis in iron homeostasis.

Taken together, *nfs1*^*xylP*^ and *nbp35*^*xylP*^ strains allowed individually switching off ISC/CIA and CIA, respectively, in order to discriminate possible iron-sensing defects originating from a deficiency in either ISC or CIA.

### Depletion of ISC but not CIA renders *A. fumigatus* blind for iron

**Fig. 3:**
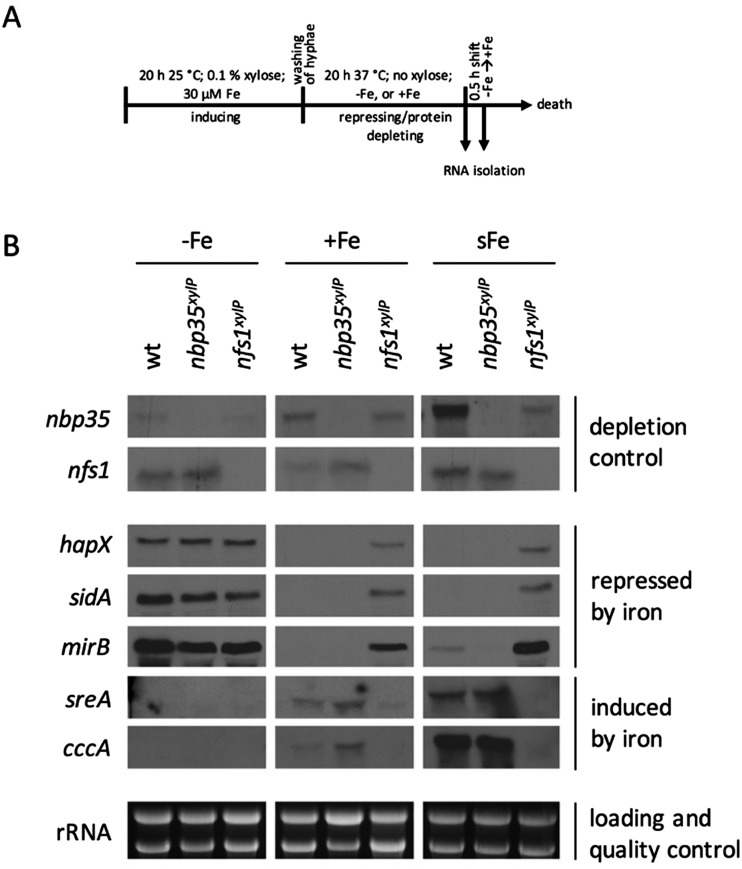
Depletion of Nfs1 causes a permanent cellular iron starvation mode. (A) Scheme of workflow for the depletion of Nfs1 and Nbp35, respectively. Strains were pre-incubated at 25 °C for 20 h in MM +Fe with 0.1% xylose prior to growth in MM without xylose for 20 h at 37 °C under iron starvation (–Fe), iron sufficiency (+Fe), and shift-iron conditions (sFe). (B) Total RNA was isolated and subject to Northern analysis examining iron responsive genes. Transcript levels of *nbp35* and *nfs1* are shown to demonstrate the degree of transcript downregulation under repressing conditions. Ribosomal RNA (rRNA) is shown as a control for RNA quality and loading.

To analyze the effects of the depletion of ISC and CIA, respectively, *nfs1*^*xylP*^ and *nbp35*^*xylP*^ strains were grown under growth-permitting inducing conditions (0.1% xylose) for 20 h at 25 °C. Subsequently, mycelia were harvested and grown for another 20 h at 37 °C under non-inducing conditions (without xylose). After this procedure, *nfs1* and *nbp35* transcript levels were reduced below the detection limit (Fig. 3B and Fig. S2, ESI†). This is comparable to the commonly used GAL system-mediated gene knockdown strategy in *S. cerevisiae*.^78^ This strategy allows an initial production of FeS to supply client proteins to support growth, while the subsequent non-inducing phase results in the depletion of FeS due to the blocking expression of the respective genes. To analyze the effect of the depletion of ISC and CIA on iron homeostasis, this set-up was performed under iron starvation (–Fe), iron sufficiency (+Fe), and shift-iron (sFe) conditions. Shift-iron represents a shift from iron starvation to iron replete conditions for 0.5 h at the end of the non-inducing growth phase (Fig. 3A). Such an experimental set-up allowed monitoring the short-term effects of iron on gene transcription. It was previously shown that the shift-iron condition results in a high induction of genes activated by iron and a downregulation of genes repressed by iron.^4^

To monitor the transcriptional response to FeS depletion, we examined the transcript levels of five genes, previously shown to be iron-regulated,^4^,^6^,^8^ by Northern blot analysis (Fig. 3B). We monitored three genes, which are upregulated during iron starvation and quickly downregulated in shift-iron conditions: *hapX* (iron-regulatory transcription factor), *mirB* (TAFC transporter), and *sidA* (siderophore biosynthesis); and two genes, which are downregulated during iron starvation and quickly upregulated during shift-iron conditions: *sreA* (iron regulatory transcription factor) and *cccA* (vacuolar iron transporter mediating iron detoxification).

The wt showed the expected transcriptional pattern of these six genes during iron starvation, iron sufficiency, and shift-iron conditions (Fig. 3B). During iron starvation, the depletion of neither Nfs1 nor Nbp35 had a significant impact on the transcript levels of the five genes. In contrast, during iron sufficiency and shift-iron conditions, the depletion of Nfs1 (required for mitochondrial and consequently also cytosolic FeS biosynthesis) but not Nbp35 (required for cytosolic FeS biosynthesis only) resulted in significantly increased transcript levels of *hapX*, *mirB*, and *sidA*, compared to wt, indicating a cellular iron starvation status. These data indicate that iron-sensing in *A. fumigatus* depends on mitochondrial but not cytosolic FeS biosynthesis. The derepression of these genes during iron sufficiency and shift-iron conditions is reminiscent of the transcriptional changes caused by lack of the transcription factor SreA,^4^ indicating that the signal from mitochondrial FeS biosynthesis is mediated by SreA.

During iron sufficiency, the depletion of Nfs1 but not Nbp35 decreased transcript levels of *sreA* and *cccA*, similar to the wt level during iron starvation, emphasizing the cellular iron-starvation status. Moreover, in shift-iron conditions, the depletion of Nfs1 but not Nbp35 blocked the induction of *sreA* and *cccA* again, indicating that the induction process depends on mitochondrial but not cytosolic FeS biosynthesis. This iron response has previously been shown to be mediated by the transcription factor HapX,^7^ suggesting that HapX requires the signal from mitochondrial FeS biosynthesis for proper function. The lack of activation of *cccA* expression caused by Nfs1-depletion most likely explains the increased susceptibility to iron toxicity of *nfs1*^*xylP*^ found in plates with limited xylose induction (Fig. 2A). Moreover, it is noteworthy that the transcription of *nbp35* itself is also repressed during iron starvation and induced during shift-iron conditions in wt cells (Fig. 3B). As previously reported,^6^*nbp35* belongs to the HapX regulon along with *cccA* and *sreA*. Similar to *cccA* and *sreA*, the induction of *nbp35* is impaired in shift-iron conditions in Nfs1-depleted cells (Fig. 3B). These data reveal that the downregulation of ISC results in a transcriptional downregulation of CIA, thus demonstrating regulatory dependence in addition to the metabolic dependence.

Remarkably, during iron sufficiency, the depletion of Nbp35 caused an upregulation of *sreA* and *cccA* compared to wt (Fig. 3B). Above, we have shown that the induction of these genes in shift-iron conditions depends on a signal originating from mitochondrial FeS biosynthesis, most likely X–S. As the depletion of Nbp35 blocks the consumption of the X–S compound by CIA, X–S is expected to accumulate to a higher degree upon Nbp35 depletion compared to wt. Therefore, the upregulation of *sreA* and *cccA* during iron sufficiency might be caused by the increase of X–S, *i.e.*, the ISC signal for iron-sensing.

### Lack of the mitochondrial iron importer MrsA impairs iron-sensing

**Fig. 4:**
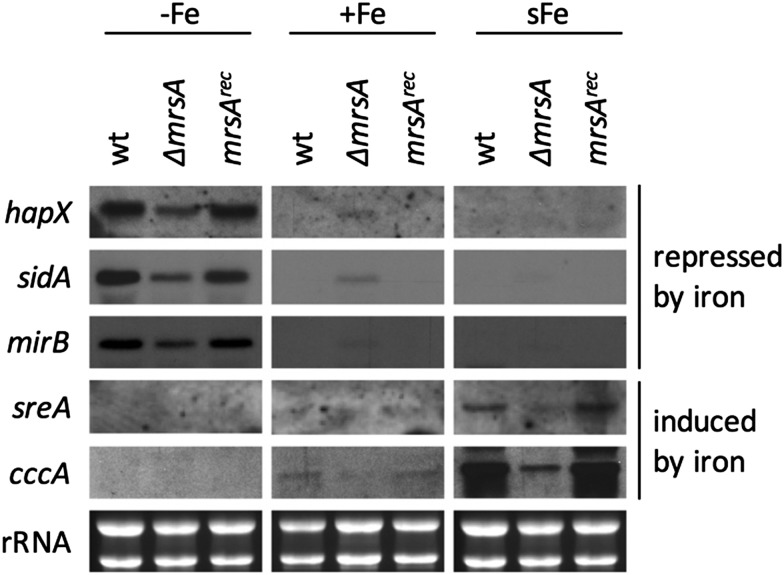
Lack of MrsA impairs iron-sensing in *A. fumigatus.* Strains were grown for 20 h at 37 °C during iron starvation (–Fe), iron sufficiency (+Fe), and subject to shift-iron conditions. Strain *mrsA*^*rec*^ is the *ΔmrsA* mutant with a reintegrated *mrsA* gene copy to assure *mrsA*-deletion specific effects. Due to the extreme growth defect of *ΔmrsA* during iron starvation conditions, the *ΔmrsA* culture contained 0.5 μM iron. This higher iron content during iron starvation is most likely responsible for the decreased expression of *hapX*, *sidA*, and *mirB* in this mutant compared to the other strains. Ribosomal RNA (rRNA) is shown as the control for RNA quality and loading.

In *A. fumigatus*, a mitochondrial iron importer MrsA has been characterized with respect to virulence and resistance against azoles and oxidative stress.^24^ MrsA is a homolog of the paralogous mitochondrial iron importers Mrs3 and Mrs4 in *S. cerevisiae*.^79^,^80^ Several essential iron-dependent pathways, including ISC, are localized in the mitochondrial matrix, and consequently mitochondrial import is expected to be essential. The viability of *A. fumigatus* mutant strains lacking MrsA and *S. cerevisiae* strains lacking Mrs3/Mrs4 indicates the presence of alternative mitochondrial iron importers. In agreement, the mitochondrial carrier protein Rim2 was shown to co-import pyrimidine nucleotides and iron in *S. cerevisiae*.^81^,^82^ Nevertheless, MrsA appears to be a major mitochondrial iron importer in *A. fumigatus* as its inactivation caused a growth defect particularly during iron starvation and iron excess.^24^ Moreover, the lack of *mrsA* was shown to upregulate genes involved in iron acquisition during iron sufficiency; however, without a mechanistic explanation.^24^ As mitochondrial iron import is essential for ISC biosynthesis, we reanalyzed the impact of a lack of MrsA on the transcriptional iron response by Northern analysis (Fig. 4). During iron limitation, a lack of MrsA had no significant influence on transcript levels of iron-regulated genes. In contrast, during iron sufficiency, a lack of MrsA upregulated transcript levels of *hapX*, *mirB*, and *sidA*. This effect was similar to the depletion of Nfs1 (Fig. 3B), but not as strong, most likely due to the presence of alternative mitochondrial iron importers, while there is no alternative for Nfs1 activity. Moreover, a lack of MrsA impaired the short-term induction of *sreA* and *cccA* during shift-iron conditions (Fig. 4), again an effect similar to Nfs1 depletion.

Taken together, these data underline the importance of mitochondria in iron-sensing and are in agreement with ISC being important for cellular iron-sensing (see above).

### Depletion of GSH leads to constant iron starvation

GSH has previously been shown to be involved in FeS biosynthesis, the coordination of FeS, and iron regulation in *S. cerevisiae*^39^ and *S. pombe*.^40^ To investigate the effect of GSH on iron regulation in *A. fumigatus*, a strain lacking γ-glutamylcysteine synthetase (*ΔgshA*) was generated. To assure the specificity of gene deletion effects, the *ΔgshA* strain was reconstituted with a functional *gshA* gene copy, yielding strain *gshA*^*rec*^. This *ΔgshA* mutant strain was unable to grow without GSH supplementation (Fig. 2B), demonstrating that GshA is indeed essential for GSH biosynthesis in *A. fumigatus*.

**Fig. 5:**
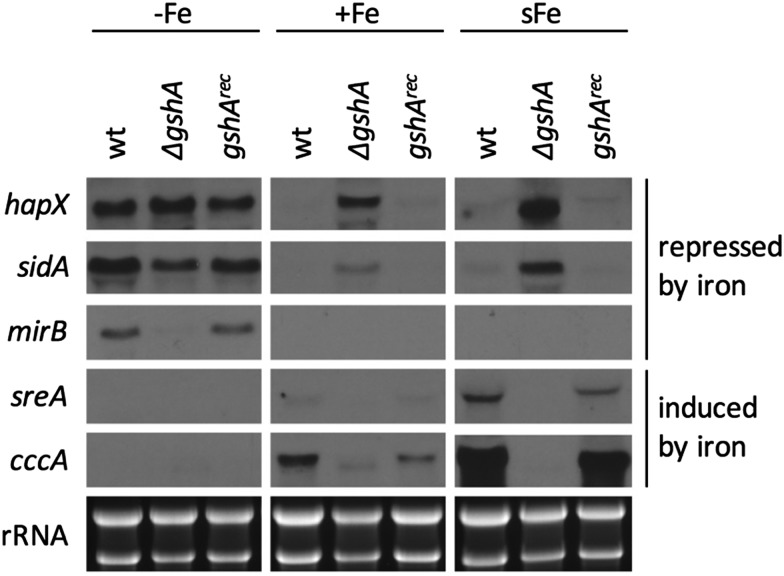
GSH depletion impairs iron-sensing in *A. fumigatus.* Strains were pre-incubated at 25 °C for 20 h in MM +Fe with 5 mM GSH prior to growth in MM without GSH for 20 h at 37 °C under iron starvation (–Fe), iron sufficiency (+Fe), and shift-iron conditions (sFe). Total RNA was isolated and subject to Northern analysis examining iron-responsive genes. Ribosomal RNA (rRNA) is shown as the control for RNA quality and loading.

Northern analysis (Fig. 5) revealed that GSH depletion resulted in similar effects as Nfs1 depletion (Fig. 3B) or MrsA inactivation (Fig. 4): the upregulation of *hapX* and *sidA* (overexposure of the Northern blot revealed also the upregulation of *mirB*; Fig. S3, ESI†) during iron sufficiency as well as the impaired short-term induction of *sreA* and *cccA* under shift-iron conditions.

Taken together, these data demonstrate that GSH is crucial for cellular iron-sensing. Mechanistically, GSH might be involved in the coordination of FeS,^44^ in *e.g.*, monothiol glutaredoxins, which are involved in the regulation of iron-responsive genes.^40^,^83^–^85^ Moreover it has been shown in *S. cerevisiae* that GSH is involved in ISC by supporting the monothiol glutaredoxin Grx5.^17^,^86^

However, in contrast to Nfs1 depletion (Fig. 3B) or MrsA inactivation (Fig. 4), GSH depletion significantly downregulated *mirB* during iron starvation and did not upregulate *mirB* during iron sufficiency (Fig. 5). Noteworthy, *mirB* activation during iron starvation was previously shown to depend highly on HapX function, significantly more than, for example, *sidA* or *hapX* itself.^6^,^7^ Therefore, these data might indicate that GSH is involved not only in the repression of genes induced by iron starvation (*sidA*, *hapX*; mediated by SreA) and the induction of genes induced by iron (*cccA*, *sreA*; mediated by HapX), but also the induction of genes induced by iron starvation (*mirB*, mediated by HapX). The fact that the depletion of Nfs1 did not affect *mirB* transcript levels during iron starvation (Fig. 3B) suggests an ISC-independent role of GSH in adaptation to iron starvation. This contrasts the situation in *S. cerevisiae* and *S. pombe*, for which GSH only has been demonstrated to be essential for the repression of iron uptake and the induction of iron-responsive genes, but not the induction of genes involved in iron uptake.^40^,^51^

### Depletion of Nfs1 and GSH as well as the inactivation of MrsA cause increased siderophore-mediated iron uptake

The results above revealed that the depletion of Nfs1 and GSH as well as the inactivation of MrsA results in the transcriptional derepression of siderophore biosynthesis (*sidA*) and siderophore-mediated iron uptake (*mirB*) during iron sufficiency. To monitor siderophore-mediated iron uptake, we grew *A. fumigatus* wt and mutant strains in liquid minimal medium with the iron loaded from TAFC (TAFC^Fe^), the major extracellular siderophore of *A. fumigatus*, as the sole iron source. TAFC exhibits a reddish colour upon the chelation of iron, which allows determining its concentration by photometric quantification. As one molecule TAFC binds 1 ferric ion, TAFC^Fe^ uptake directly corresponds to iron uptake. TAFC^Fe^ uptake was determined indirectly by the quantification of TAFC^Fe^ depletion of the culture supernatant after 24 h of growth and with normalization to the biomass.

**Fig. 6:**
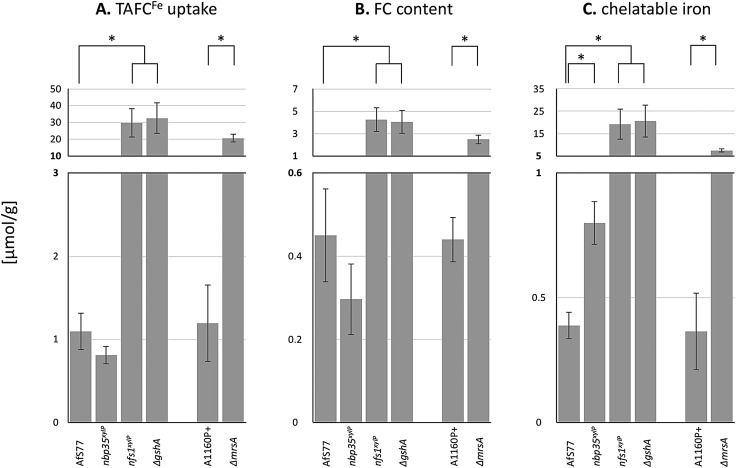
Depletion of Nfs1, Nbp35, or GSH as well as the inactivation of MrsA affects TAFC^Fe^ uptake (A), cellular FC^Fe^ accumulation (B), and/or the cellular chelatable iron pool (C). Fungal strains were pre-incubated at 25 °C for 20 h in MM +Fe with xylose (strains *nbp35*^*xylP*^, *nfs1*^*xylP*^) or GSH (Δ*gshA*). For the depletion, mycelia were washed and growth was continued in MM with 100 μM TAFC^+Fe^ but without xylose or GSH for 20 h at 37 °C. AfS77 is the wt genetic background strain for *nbp35*^*xylP*^, *nfs1*^*xylP*^, and Δ*gshA*; A1160P+ is the wt genetic background strain for Δ*mrsA.* Experiments were carried out in triplicate. Means ± SD are shown. Moreover, values are given in Table S4 (ESI†). Asterisks indicate significant differences (*p*-values < 0.01; unpaired two-tailed *t*-test).

*A. fumigatus* wt mycelia consumed about 1 μmol of iron per gram dry weight (Fig. 6A), which matches the reported mycelial iron content of *Aspergillus nidulans*^87^ and *A. fumigatus* in previous studies.^4^ In agreement with the transcriptional data, the depletion of Nfs1 and GSH as well as the inactivation of MrsA resulted in a significantly increased uptake (about 30-fold upon depletion of Nfs1 or GshA, about 18-fold for Δ*mrsA*) of iron (Fig. 6A). In contrast, the depletion of Nbp35 did not significantly impact iron uptake compared to wt (Fig. 6A). In agreement, siderophore-mediated iron uptake was not deregulated at the transcriptional level (Fig. 3B).

Taken together, these physiological data highly support and substantiate the data from gene expression analysis, indicating that iron regulation in the filamentous fungus *A. fumigatus* depends on mitochondrial ISC but not cytosolic CIA.

### Depletion of Nfs1, Nbp35, or GSH as well as inactivation affects the cellular iron distribution

*A. fumigatus* can store iron as the intracellular siderophore ferricrocin,^88^ termed FC^Fe^, or in the vacuole for detoxification.^8^ Moreover, iron is found complexed in proteins with or without co-factors (heme, FeS) or in the labile iron pool (LIP). To characterize the fate of consumed TAFC^Fe^ in the mycelia of wt and mutant strains, we quantified the amounts of FC^Fe^ and chelatable iron (Fig. 6B and C). The depletion of Nfs1 or GSH as well as the inactivation of MrsA resulted in a significant increase (about 10-fold upon depletion of Nfs1 or GshA, about 5-fold for Δ*mrsA*) in the cellular FC^Fe^ content. In contrast, this was not observed upon Nbp35 depletion.

In a next step, we quantified the chelatable iron pool after the separation of FC^Fe^, using bathophenanthrolinedisulfonic acid (BPS; Fig. 6C). BPS is a ferrous iron (Fe^2+^)-specific chelator, which turns reddish upon binding iron, allowing quantification by photometric measurement.^89^ The depletion of Nfs1 or GSH as well as the inactivation of MrsA resulted in a significant increase (about 50-fold upon the depletion of Nfs1 or GshA, about 18-fold for Δ*mrsA*) of the chelatable iron pool.

Taken together, all the mutants showing increased TAFC^Fe^ uptake displayed an increase in both cellular FC^Fe^ content and cellular chelatable iron pool. The repression of TAFC biosynthesis and TAFC^Fe^ uptake during iron sufficiency is mediated by SreA^4^ and the genetic inactivation of SreA has previously been shown to result in an increased cellular iron content accompanied by an increased FC^Fe^ content.^4^ The similarity of the effects of SreA inactivation and the depletion of Nfs1 or GSH as well as the inactivation of MrsA suggests that ISC generates the signal for sensing iron by SreA. The excessive uptake and cellular accumulation of iron is in line with the observed decrease in iron resistance (Fig. 2A). The decreased iron resistance is most likely related to the increased levels of chelatable iron, as the amount of “free” chelatable iron has been reported to trigger the production of toxic hydroxyl radicals.^90^,^91^ Notably, the depletion of Nfs1 or GSH caused a significantly higher increase in iron uptake and cellular iron accumulation compared to the inactivation of MrsA (Fig. 6A). This is most likely due to the fact that *A. fumigatus* possesses MrsA-independent mitochondrial iron import.^81^ Consequently, MrsA inactivation blocks ISC biosynthesis only partly in contrast to Nfs1 depletion.

The depletion of Nbp35 did not increase either TAFC^Fe^ uptake or cellular FC^Fe^ accumulation. Nevertheless, it increased the chelatable iron pool by about 2-fold, indicating a defective cellular iron distribution. The assembly of [4Fe–4S] clusters on Nbp35 and Cfd1 is considered as the first step in CIA and its function highly depends on Nfs1 activity for the generation of factor X–S.^17^,^28^,^75^ Thereby Nbp35 requires X–S as a regulatory signal or as a substrate for [4Fe–4S] cluster assembly in CIA. Consequently, the depletion of Nbp35 might lead to an accumulation of X–S as it is not further processed. Combining this possible X–S tailback and the finding that Grx3/4 binds increased amounts of iron upon Nbp35 or Dre2 depletion,^37^ this enigmatic compound X–S either provokes the binding of free iron on Grx3/4, the *de novo* formation of FeS on Grx3/4, or might be already an FeS, which is produced in mitochondrial ISC. Grx3/4 is a monothiol glutaredoxin, which coordinates a [2Fe–2S] cluster in a GSH-dependent manner.^42^,^43^,^92^ It has been suggested that X–S is [2Fe–2S] coordinated by GSH as this complex represented *in vitro* a substrate for the mitochondrial exporter Atm1,^36^ which transports the compound X–S to the cytoplasm.^75^ On the other hand, the transport rate and efficiency of transport of GSH-coordinated [2Fe–2S] by Atm1 was adjudged to be rather low.^17^ Nevertheless, it has also been suggested that Nbp35 might assemble [4Fe–4S] clusters from mitochondrial [2Fe–2S] clusters in *Arabidopsis thaliana*.^93^

Whatever the chemical nature of X–S is, the consequences of its accumulation upon Nbp35 depletion, which blocks CIA and the incorporation of [4Fe–4S] into cytosolic proteins, most likely causes the increased chelatable iron pool. The increased chelatable iron pool might give reason for the decreased iron resistance of the *nbp35*^*xylP*^ mutant strain found in plate assays (Fig. 2A).

### Depletion of both Nfs1 or Nbp35 causes decreased resistance to phleomycin and hydrogen peroxide

An increase in the chelatable pool causes oxidative stress *via* the Haber–Weiss/Fenton reaction and consequently decreased resistance to oxidative stressors, such as hydrogen peroxide.^91^ Moreover, an increased chelatable iron pool causes decreased resistance to phleomycin, which interacts with intracellular ferrous iron to form reactive oxygen species, resulting in genotoxicity.^94^–^97^ Previously, the deregulation of iron uptake due to the lack of the iron regulator SreA has been shown to cause decreased phleomycine resistance in *A. nidulans* and *A. fumigatus*.^4^,^94^ Our results demonstrated that the depletion of both Nfs1 and Nbp35 resulted in an increase of the chelatable iron pool, although by different means (Fig. 6). The depletion of both Nfs1 and Nbp35 decreased the minimal inhibitory concentration (MIC) of hydrogen peroxide and phleomycin (Table 1), supporting increased levels of the cellular chelatable iron pool.

**Table 1:** Depletion of Nfs1 or Nbp35 causes decreased resistance to phleomycin and hydrogen peroxide. Resistance was determined with serial dilution MIC testing under low _*p*_*xylP* inducing conditions (minimum required for growth; 0.02% xylose for *nbp35*^*xylP*^ or 0.03% for *nfs1*^*xypP*^) as described in the experimental section. Noteworthy, under high _*p*_*xylP* inducing conditions, both *nbp35*^*xlyP*^ and *nfs1*^*xylP*^ showed wt-like resistance to both stressors

Strain	Phleomycin [μg mL^–1^]	H_2_O_2_ [mM]
wt	64	16
*nbp35* ^*xlyP*^	32	8
*nfs1* ^*xylP*^	32	8

## Conclusions

This study demonstrates that in *A. fumigatus*, the signal for iron-sensing is generated in mitochondria by ISC, while cytosolic CIA is dispensable for correct iron regulation. Likewise, ISC was found to be crucial for the repression of iron uptake not only in *S. cerevisiae* but also in *C. albicans*, *S. pombe*, and *C. neoformans.*^52^–^55^ The importance of ISC in iron-sensing is underlined by the fact that decreased mitochondrial iron import due to inactivation of the mitochondrial iron importer MrsA causes similar effects as ISC inactivation, *i.e.*, transcriptional deregulation of iron metabolism, increased siderophore-iron uptake, and an increased chelatable iron pool. Similarly, a deficiency of *mrsA* homologs in *C. albicans*, *S. cerevisiae*, and *C. neoformans* leads to a de-repression of iron uptake.^80^,^98^,^99^

Nevertheless, the role of CIA in iron regulation was not analyzed in these species except for *S. cerevisiae*, in which CIA is dispensable for correct iron-sensing. For *A. fumigatus* (as the first organism apart from *S. cerevisiae*), we demonstrated that CIA, at least the essential CIA component Nbp35, is not required for signaling iron sufficiency, *i.e.*, the repression of siderophore-mediated iron acquisition, which has previously been shown to be mediated by the transcription factor SreA.^4^ SreA homologs possess four conserved cysteine residues in between the GATA-type zinc fingers.^94^ The SreA homolog of *S. pombe*, Fep1, has been demonstrated to coordinate iron or FeS depending on the presence of these conserved cysteine residues when expressed recombinantly in *Escherichia coli*, and, moreover, these cysteine residues were shown to be essential for Fep1 function.^13^,^40^ Similarly, the recombinant SreA homolog from *Pichia pastoris* was shown to coordinate a [2Fe–2S] cluster with the four conserved cysteines,^100^ while the recombinant SreA homolog of *Neurospora crassa* exhibited a reddish color depending on the presence of the conserved four cysteine residues.^101^ In agreement with a function in iron-sensing of the *N. crassa* SreA homolog, the site-directed mutagenesis of these conserved cysteines impaired iron-sensing. Taken together, these data strongly indicate that SreA homologs sense iron by the binding of FeS.

Moreover, this study demonstrated that ISC is crucial for activation of iron-dependent pathways in response to iron availability, which was previously shown to be mediated by the iron-regulatory transcription factor HapX.^7^ It has been suggested previously that Php4, the *S. pombe* homolog of HapX, is inhibited during iron sufficiency in a [2Fe–2S] cluster-dependent manner.^83^,^102^ In contrast to *A. fumigatus* HapX, a function of *S. pombe* Php4 has not been reported under iron excess conditions. This indicates a different mechanism for HapX iron-sensing, which not only includes the inactivation of the iron starvation response, but additionally the activation of the high iron response. Several indications suggest that the binding of FeS might be a plausible trigger for this switch: HapX comprises several cysteine-rich regions (CRR), whereby two of these CRR were shown to be essential for the activating function of HapX during iron excess.^7^ One of the CRR contains a motif (CGFC) showing similarity to the domain coordinating [2Fe–2S] in cytosolic monothiol glutaredoxin, termed Grx3/4 in *S. cerevisiae*. Moreover, this CRR shows high similarity to a domain in *S. cerevisiae* Yap5 (Fig. S4, ESI†). Similar to HapX in *A. fumigatus*, Yap5 is essential for activation of the iron-dependent pathways in response to iron, including the transport of iron into vacuoles.^103^ In contrast to HapX, however, Yap5 has no function during iron starvation. Yap5 function was shown to depend on ISC but not CIA^50^ and the CRR-similar domain was shown to be essential for Yap5 function as well as to coordinate a [2Fe–2S] cluster. The regulation of *S. cerevisiae* iron uptake during iron starvation is performed by the transcription factors Aft1/2. It has been shown that the inactivation of the Aft1/2-mediated iron starvation response under iron replete conditions depends on ISC and on the coordination of [2Fe–2S] cluster, but not on CIA.^48^,^51^

Furthermore, this study indicated that GSH is not only essential for the repression of iron uptake and induction of iron responsive genes, as reported in *S. cerevisiae* and *S. pombe*,^40^,^51^ but additionally plays a role in the induction of genes involved in iron uptake. This has not been reported before and might be specific for *Aspergillus*. HapX, which plays a role in the activation of these genes, contains a motif (CGFC) showing similarity to the domain coordinating, together with GSH, [2Fe–2S] in cytosolic monothiol glutaredoxin (see above).^7^ Therefore, it is tempting to speculate that GSH is involved in HapX-mediated regulation.

Interestingly, our results demonstrated that not only the depletion of ISC, which causes increased iron acquisition, but also the depletion of CIA, which does not significantly affect iron uptake, results in an increase in the chelatable iron pool, although by different means (Fig. 6). The latter effect has not been observed before.

Taken together, this study characterized iron homeostasis maintenance in a siderophore-producing fungal species and suggests that HapX and SreA might also sense [2Fe–2S] clusters, the biosynthesis of which depend on ISC but not on CIA.

## Abbreviations

BPS: Bathophenanthrolinedisulfonate
CIA: Cytoplasmic iron–sulfur cluster assembly
FeS: Iron–sulfur cluster
ISC: Core iron–sulfur cluster assembly machinery
–Fe: Iron starvation
+Fe: Iron sufficiency
sFe: Shift-iron
hFe: High-iron
GSH: Glutathione

## Conflicts of interest

There are no conflicts of interest to declare.

## Supplementary Material

Supplementary information
