# Supplementary material for: Iron-sensing is governed by mitochondrial, not by cytosolic iron–sulfur cluster biogenesis in Aspergillus fumigatus
Source: Metallomics. 2018 Nov 5;10(11):1687–700. doi: 10.1039/c8mt00263k (PMC6250123; doi:10.1039/c8mt00263k)
Supplement: Supplementary file 1 [file MT-010-C8MT00263K-s001.pdf]

Electronic Supplementary Material (ESI) for Metallomics.
This journal is © The Royal Society of Chemistry 2018

Supplementary Information

Supplementary Table S1: Strains used in this study: $_{p}$*xylP* is the promoter region of the 1,4-endoxylanase (*xylP*) promoter. The asterisk (*) indicates a second *pyrG* gene copy (endogenous *A. fumigatus pyrG*); both copies are not functional.

Strain	Background	Genotype	Reference
AfS77 (wt)	ATCC46645	*ΔakuA::loxP*	[1]
nbp35^{xylP}	AfS77	$_{p}$*nbp35::hph-*$_{p}$*xylP*	this study
nfs1^{xylP}	AfS77	$_{p}$*nfs1::hph-*$_{p}$*xylP*	this study
ΔgshA	AfS77	*ΔgshA::ptrA*	this study
gshA^{rec}	AfS77	*ΔgshA::ptrA, 5'gshA::gshA-hph*	this study
A1160	CEA17	*ΔakuB::pyrG*$^{-}$*, pyrG*$^{-}$ *	[2]
A1160P+ (wt)	A1160	*ΔpyrG*$^{-}$*::pyrG*$^{+}$	[3]
ΔmrsA	A1160	*ΔmrsA::pyrG*$^{+}$	[4]

Supplementary Table S2: Primers used in this study:

Number	Primer	Sequence
1	nbp35_5'f	AATTCGAGCTCGGTACGTTGCATCTAATACTACATTTC
2	nbp35_5'r	TACCTAGGTATTATGCGGAAACCAGC
3	hph1_f	GCATAATACCTAGGTACAGAAGTCC
4	hph1_r	CGCATCAGTGCGGCCTCTAGAAAGAAGGATTACC
5	xylP1_f	ATCCTTCTTTCTAGAGGCCGCACTGATGCGAGCAACAGTATG
6	xylP1_r	GGCGCCATGGTTGGTTCTTCGAGT
7	nbp35_3'f	AACCAACCATGGCGCCGTCCGTG
8	nbp35_3'r	GCCAAGCTTGCATGCCGGGCTGTCCGGGAAG
9	nfs1_5'f	AATTCGAGCTCGGTACAAGAGTGATATTAGAGCGAA
10	nfs1_5'r	TACCTAGGAATTTAGATGTTGCTCAACC
11	hph2_f	TCTAAATTCCTAGGTACAGAAGTCC
12	xylP2_r	CTAGACATGGTTGGTTCTTCGAGT
13	nfs1_3'f	AACCAACCATGTCTAGCGTTACGC
14	nfs1_3'r	GCCAAGCTTGCATGCCTGCGTCGAACATAGCA
15	gshA_5'f	CACGGTGTTGAGTTGGTC
16	gshA_5'r	ATCCTGCAGGACCCAACCAACCCAGTA
17	gshA_3'f	GCTACCTACCTTGACCTC
18	gshA_3'r	CCTCTTGACGGGAATCTG
19	gshA_5'nest	TCCGCTTCCTGTCTTTCC
20	gshA_3'nest	TTCTACACCAGCACCTCC
21	gshA_f	AATTCGAGCTCGGTACTTTGTGAGAGACCAGC
22	gshA_r	TACTCGAGGATCAGTTTGGTTACG
23	hph4_f	AACTGATCCTCGAGTACCATTTAATTCT
24	hph4_r	GCCAAGCTTGCATGCCACGCGTTTTATTCTTGTT

Supplementary Table S3: Homologies between *S. cerevisiae*, *A. fumigatus* and *H. sapiens.* *S. cerevisiae* proteins were used as bait to search the *A. fumigatus* and human proteoms (NCBI-BLAST on FungiDB[5,6]).

***S. cerevisiae* , strain S288C**		***A. fumigatus* A1163 homolog**	**BLASTP *A. fumigatus***		***H. sapiens***
**Name**	**ID**	**ID**	**E-value**	**Identity**	
MRS3	YJL133W	AFUB_078550	1,00E-92	156/303 (51%)	SLC25A37
MRS4	YKR052C	AFUB_078550	4,00E-86	149/307 (49%)	SLC25A37
YFH1	YDL120W	AFUB_067610	1,00E-26	50/117 (43%)	FXN
NFS1/SPL1	YCL017C	AFUB_034990	0	311/474 (66%)	NFS1
ISD11	YER048W-A	AFUB_042560	1,00E-16	34/80 (43%)	LYRM4
ARH1	YDR376W	AFUB_028270	3,00E-86	197/533 (37%)	FDXR
YAH1	YPL252C	AFUB_052400	4,00E-61	85/133 (64%)	FDX2
ISU1/NUA1	YPL135W	AFUB_063840	5,00E-63	91/119 (76%)	ISCU
SSQ1/SSH1/SSC2	YLR369W	AFUB_025800	0	326/626 (52%)	HSPA9
JAC1	YGL018C	AFUB_082890	1,00E-27	56/177 (32%)	HSCB
MGE1/YGE1/GRPE	YOR232W	AFUB_028670	1,00E-49	97/198 (49%)	GRPEL1
GRX5	YPL059W	AFUB_063050	3,00E-57	88/144 (61%)	GLRX5
ISA1	YLL027W	AFUB_067770	4,00E-56	81/117 (69%)	ISCA1
ISA2	YPR067W	AFUB_026180	2,00E-22	50/126 (40%)	ISCA2
NFU1/NUB1	YKL040C	AFUB_005020	3,00E-50	97/235 (41%)	NFU1
IBA57/CAF17	YJR122W	AFUB_060100	2,00E-21	127/492 (26%)	IBA57
BOL3/AIM1	YAL046C	AFUB_078500	1,00E-11	24/70 (34%)	BOLA3
CFD1/DRE3	YIL003W	AFUB_052120	3,00E-94	152/306 (50%)	NUBP2
NBP35	YGL091C	AFUB_031640	9,00E-161	210/313 (67%)	NUBP1
DRE2	YKR071C	AFUB_008090	2,00E-28	53/119 (45%)	CIAPIN1
TAH18	YPR048W	AFUB_054840	8,00E-102	227/687 (33%)	NDOR1
GRX3	YDR098C	AFUB_030610	8,00E-68	107/256 (42%)	GLRX3
GRX4	YER174C	AFUB_030610	5,00E-67	110/259 (42%)	GLRX3
NAR1	YNL240C	AFUB_068950	2,00E-73	172/490 (35%)	NARFL
CIA1	YDR267C	AFUB_008350	3,00E-64	146/427 (34%)	CIAO1
CIA2	YHR122W	AFUB_083590	5,00E-51	80/131 (61%)	FAM96B
MET18/MMS19	YIL128W	AFUB_082160	1,00E-50	267/1113 (24%)	MMS19

Supplementary Table S4: Numerical values [µmol/g dry weight] for iron uptake, FC- and chelatable iron content

	TAFC uptake		FC content		chelatable iron	
	mean	standard deviation	mean	standard deviation	mean	standard deviation
**AfS77**	1.10	0.22	0.45	0.11	0.39	0.05
nbp35 ^{xylP}	0.82	0.10	0.30	0.09	0.80	0.09
nfs1 ^{xylP}	29.87	8.38	4.27	1.07	19.27	6.72
ΔgshA	32.63	9.15	4.07	1.02	20.63	7.07
**A1160P+**	1.20	0.46	0.44	0.05	0.37	0.15
ΔmrsA	20.67	2.29	2.50	0.40	7.40	0.72

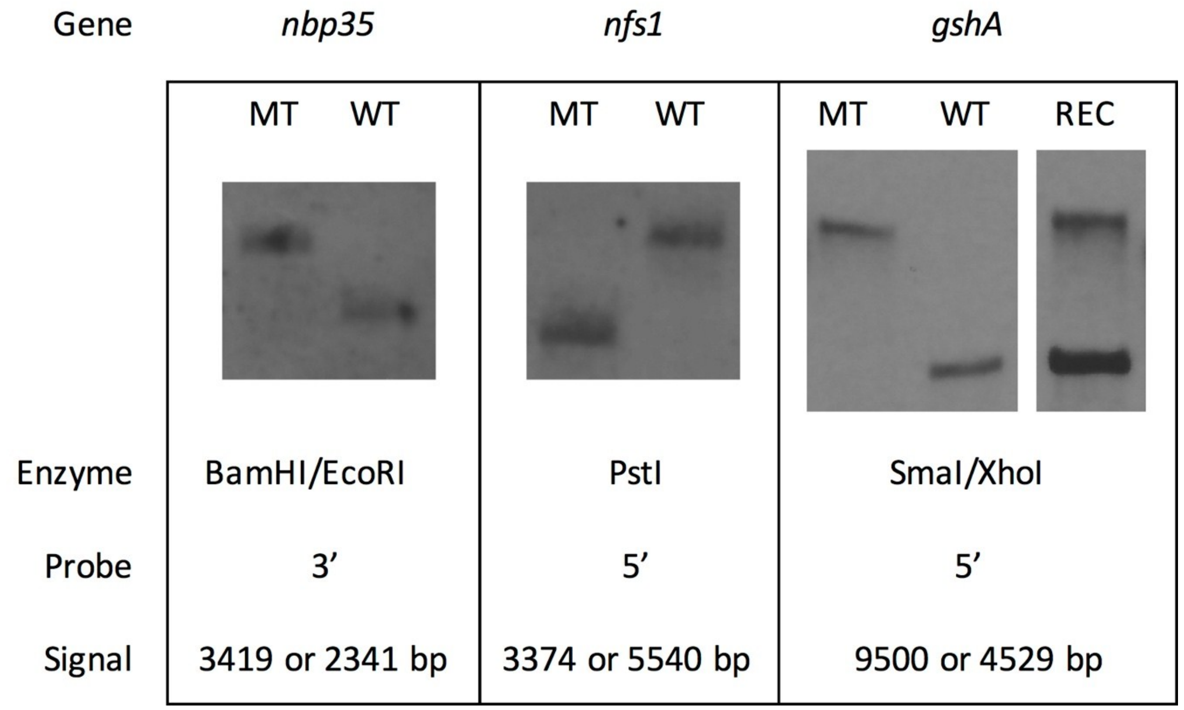

Supplemental Fig. S1: Southern blot confirmed correct gene manipulation. Target genes are indicated at the top. WT is the recipient strain; MT is the manipulated mutant strain. Enzyme indicates the restriction enzyme(s) used to digest genomic DNA. Probe indicates if the hybridization probe targeted 5’- or 3’ homologous region used for homologous recombination. Signal specifies the length of the expected fragments for WT/MT.

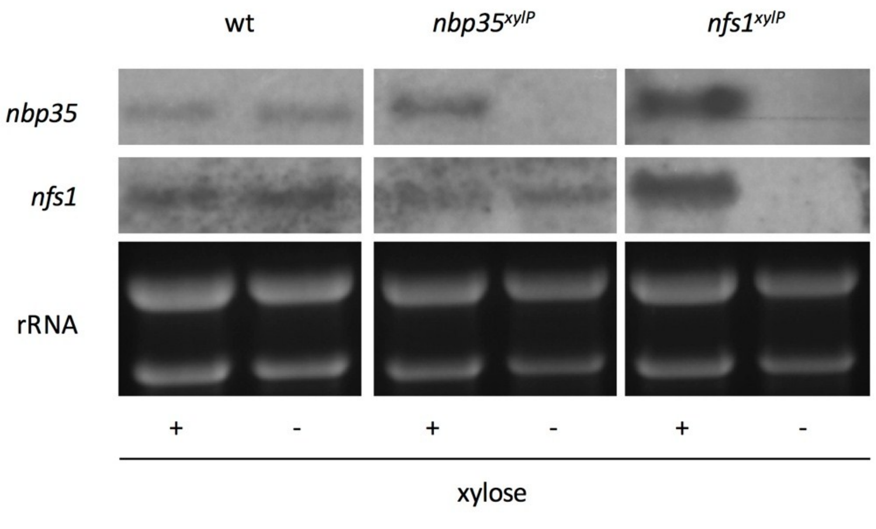

Supplemental Fig. S2: Xylose-dependent transcription of $_{p}$*xylP*-targeted genes. Strains were cultivated as described in the experimental part for 20 hours in MM +Fe 0.1% xylose 25°C. After washing the germlings one half from each strain was added to MM +Fe 0.1% xylose, the other half to MM +Fe and no xylose for 20 hours at 37°C. Expression levels of *nbp35* and *nfs1* were compared at different conditions by performing Northern analysis.

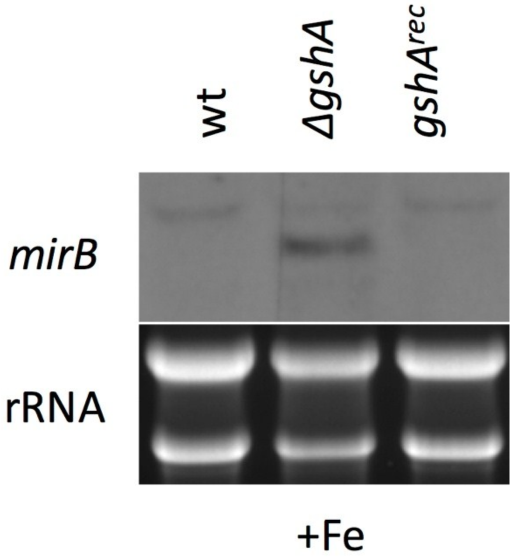

Supplemental Fig. S3: GSH depletion derepresses *mirB* during iron sufficiency. In contrast to Fig. 5, the film for detection of signals was exposed longer. Ribosomal RNA (rRNA; same as in Fig. 5) is shown as control for RNA quality and loading.

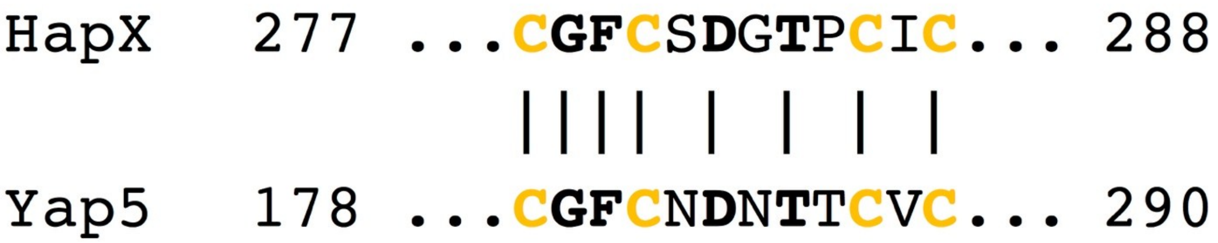

Supplemental Fig. S4: Alignment of the cysteine-rich regions of HapX and Yap5.

References:

1 T. Hartmann, M. Dümig, B. M. Jaber, E. Szewczyk, P. Olbermann, J. Morschhäuser and S. Krappmann, Validation of a self-excising marker in the human pathogen Aspergillus fumigatus by employing the beta-rec/six site-specific recombination system, *Appl. Environ. Microbiol.*, 2010, **76**, 6313–7.

2 M. E. da Silva Ferreira, I. Malavazi, M. Savoldi, A. A. Brakhage, M. H. S. Goldman, H. S. Kim, W. C. Nierman and G. H. Goldman, Transcriptome analysis of Aspergillus fumigatus exposed to voriconazole, *Curr. Genet.*, 2006, **50**, 32–44.

3 M. G. Fraczek, M. Bromley, A. Buied, C. B. Moore, R. Rajendran, R. Rautemaa, G. Ramage, D. W. Denning and P. Bowyer, The cdr1B efflux transporter is associated with non-cyp51a-mediated itraconazole resistance in Aspergillus fumigatus, *J. Antimicrob. Chemother.*, 2013, **68**, 1486–1496.

4 N. Long, X. Xu, H. Qian, S. Zhang and L. Lu, A Putative Mitochondrial Iron Transporter MrsA in Aspergillus fumigatus Plays Important Roles in Azole-, Oxidative Stress Responses and Virulence, *Front. Microbiol.*, 2016, **7**, 1–15.

5 E. Basenko, J. Pulman, A. Shanmugasundram, O. Harb, K. Crouch, D. Starns, S. Warrenfeltz, C. Aurrecoechea, C. Stoeckert, J. Kissinger, D. Roos and C. Hertz-Fowler, FungiDB: An Integrated Bioinformatic Resource for Fungi and Oomycetes, *J. Fungi*, 2018, **4**, 39.

6 J. E. Stajich, T. Harris, B. P. Brunk, J. Brestelli, S. Fischer, O. S. Harb, J. C. Kissinger, W. Li, V. Nayak, D. F. Pinney, C. J. Stoeckert and D. S. Roos, FungiDB: an integrated functional genomics database for fungi, *Nucleic Acids Res.*, 2012, **40**, D675–D681.
